# The Interpretation of Disjunction in the Scope of *Dou* in Child Mandarin

**DOI:** 10.3389/fpsyg.2020.609492

**Published:** 2020-12-15

**Authors:** Shasha An, Peng Zhou, Stephen Crain

**Affiliations:** ^1^Center for Linguistics and Applied Linguistics, Faculty of English Language and Culture, Guangdong University of Foreign Studies, Guangzhou, China; ^2^Department of Foreign Languages and Literatures, Tsinghua University, Beijing, China; ^3^Department of Linguistics, Macquarie University, Sydney, NSW, Australia

**Keywords:** child Mandarin, disjunction, *wh*-words, *dou*, free choice items, negative polarity items

## Abstract

A recent theory provides a unified cross-linguistic analysis of the interpretations that are assigned to expressions for disjunction, Negative Polarity Items, Free Choice Items, and the non-interrogative uses of *wh*-phrases in languages such as Mandarin Chinese. If this approach is on the right track, children should be expected to demonstrate similar patterns in the acquisition of these linguistic expressions. Previous research has found that, by age four, children have acquired the knowledge that both the existential indefinite *renhe* “any” and *wh*-words in Mandarin Chinese are interpreted as Negative Polarity Items when they are bound by downward entailing operators, but the same expressions are interpreted as Free Choice Items (with a conjunctive interpretation) when they are bound by deontic modals (Mandarin *keyi*) or by the Mandarin adverbial quantifier *dou* “all”. The present study extends this line of research to the Mandarin disjunction word *huozhe*. A Truth Value Judgment Task was used to investigate the possibility that disjunction phrases that are bound by the adverbial quantifier *dou* generate a conjunctive interpretation in the grammars of Mandarin-speaking 4-year-old children. The findings confirmed this prediction. We discuss the implications of the findings for linguistic theory and for language learnability.

## Introduction

Recently a theory has been advanced that provides a unified semantic analysis of disjunction, Negative Polarity Items (NPIs), Free Choice Items (FCIs), and the non-interrogative interpretations of *wh*-phrases across languages (Fox, 2007; Chierchia, 2013). The present study tests the unification account directly, by asking if Mandarin disjunctive phrases evoke conjunctive inferences when they are bound by the quantificational adverb (hereafter, Q-adverb) *dou*. This possibility is investigated in an experimental study with Mandarin-speaking children and adults. The main finding confirms the prediction, and provides circumstantial support for the unified account.

We begin with a brief tour of the semantics of existential expressions, so-called ∃–items. Three English ∃–items, the existential indefinite *some*, the disjunction word *or*, and the polarity sensitive expression *any*, are illustrated in examples (1) - (4).

(1) *Someone* coughed.
(2) *Ted or Gen* coughed.
(3) Sally didn’t hear *anyone* cough.
(4) If Sally hears *someone*/*Ted or Gen/anyone* cough, she breaks out the cold medicine.

Sentence (1), *someone coughed*, is true if and only if there exists an individual x such that x coughed. In a domain with just two individuals, Ted and Gen, (1) is logically equivalent to the disjunctive statement in (2), *Ted or Gen coughed* (e.g., Crain and Khlentzos, 2010). The polarity sensitive expression *any* is another ∃–item (e.g., Klima, 1964; Baker, 1970; Ladusaw, 1979, 1980; Carlson, 1980; Linebarger, 1987; Horn, 1989; Krifka, 1995; among others). Sentence (3) *Sally didn’t hear anyone cough* is true only if there does not exist an individual x such that Sally heard x cough. In the domain with two individuals, Ted and Gen, this statement is true only if Sally didn’t hear Ted cough and didn’t hear Gen cough^1^. Example (4) illustrates the logical equivalence under discussion. In a domain with just two individuals, all three of the ∃–items we have discussed are licensed in the antecedent of a conditional statement.

In addition to their use as existential expressions (∃–items), the English disjunction word *or* and the polarity sensitive expression *any* also generate free choice/conjunctive inferences in certain linguistic contexts (for discussion of *or*, see Kamp, 1973; Zimmermann, 2000; Geurts, 2005; Fox, 2007; Barker, 2010; Chierchia, 2013; for discussion of *any*, see Quine, 1960; Vendler, 1967; Horn, 1972; Lasnik, 1972; Ladusaw, 1979; Carlson, 1981; Dayal, 1998). Both the disjunction word *or* and the polarity sensitive expression *any* license free choice inferences when they appear in the scope of a deontic modal, such as English *may* (Mandarin *keyi*) (Lasnik, 1972; Kamp, 1973; Lee and Horn, 1994; Dayal, 1995; Zimmermann, 2000; Geurts, 2005; Barker, 2010; Zhou et al., 2013; Huang and Crain, 2014, 2020). This is illustrated in (5) and (6).

(5) Kung Fu Panda may drive the green car *or* the orange car.
(6) Kung Fu Panda may drive *any* of the cars.

Sentences in (5) and (6) both convey the message that Kung Fu Panda (hereafter, KFP) is free to choose among the available cars. According to the unified account, the disjunction word *or* and the polarity expression *any* are initially analyzed as ∃–items in sentences as (5) and (6). The fact that these sentences generate free choice/conjunctive inferences is due to a process called recursive exhaustification, which we discuss next.

### Recursive Exhaustification

The theoretical proposal by Fox (2007), Chierchia (2013) attempts to provide a unified analysis of ∃–items, all of which give rise to free choice/conjunctive inferences via recursive exhaustification. In the following we demonstrate how the free choice reading of disjunction is derived via recursive exhaustification.

(7) KFP may drive the green car or the orange car.

The sentence in (7) can be paraphrased as a conjunctive statement: *KFP may push the green car and KFP may push the orange car* (for discussion, see Kamp, 1973; Geurts, 2005; Fox, 2007; Barker, 2010; Chierchia, 2013; Zhou et al., 2013; Zimmermann, 2000). We will render the meaning of “may” symbolically using the possibility operator, ♢. The generalization that disjunctive statements yield conjunctive truth conditions is represented by the inference pattern in (8).

(8) ♢(p ∨ q) ∼ > ♢p ∧♢q

The fact that the inference in (8) is legitimate is surprising, because a plain disjunctive sentence, i.e., one without a modal, never conveys the corresponding conjunctive inference. In fact, it typically conveys its negation. That is, from the statement *KFP pushed the green car or the orange car*, it does not follow that KFP pushed both cars. An explanation for the inference in (8) has been advanced by Fox (2007) and by Chierchia (2013). We will follow Chierchia’s formulation of the account, which involves a recursive application of an algorithm akin to that of a scalar implicature. The algorithm is referred to as recursive exhaustification because an exhaustivity (ONLY) operator is applied to its own output.

As in a typical scalar implicature, the algorithm compares the statement made by a speaker with alternative statements that the speaker might have made. According to the algorithm in question, however, these alternative statements are enriched by the exhaustivity operator, to include their associated (negative) inferences. These are inferences that would have been attributed to the speaker if these alternative statements had been produced, instead of the actual statement. At the first step in the algorithm, then, the exhaustivity operator generates inferences that enrich the alternatives to what the speaker said. Then, the exhaustivity operator (ONLY) applies a second time, in order to eliminate those enriched alternatives that are stronger than what the speaker actually said. Having sketched the general idea, we will now provide a brief overview of the two steps involved in recursive exhaustification, using the disjunctive statement in (7), repeated here as (9).

(9) KFP may drive the green car or the orange car. ♢(p ∨ q)

At the first step, the assertion is compared to its “subdomain” alternatives. These subdomain alternatives are formed using these disjuncts in the predicate phrase of the original assertion, as shown in (10) and (11). These subdomain alternatives are compared to the assertion at the second step in the algorithm, but only after they have been enriched with their associated inferences.

(10) KFP may drive the green car. subdomain alternative = ♢p.
(11) KFP may drive the orange car. subdomain alternative = ♢q.

What are the inferences associated with (10) and (11)? When a speaker asserts (9), the question under discussion is which cars KFP has been given permission to drive. The green car and the orange car are the relevant alternatives. Suppose that the speaker had asserted one of the subdomain alternatives, (10) or (11), instead of (9). If the speaker had asserted (10), this would have invited the inference in (12). Similarly, from the subdomain alternative in (11), we would have inferred (13).

(12) KFP may drive the green car, but not the orange car. ♢p ∧¬♢q
(13) KFP may drive the orange car, but not the green car. ♢q ∧¬♢p

The intuition of such inferences is as follows. Suppose your friend Mary asks *who coughed* in a situation with only two individuals, Ted and Gen. Another friend, Bruce, answers - *Ted*. We interpret Bruce’s answer as shorthand for *Only Ted coughed*. That is, we infer from Bruce’s fragment answer, *Ted*, that Gen did not cough. To account for this kind of inference, we posit that an implicit exhaustivity operator, ONLY, is operative in such discourse sequences that include disjunctive statements. This exhaustivity operator is responsible for generating the pragmatic inferences that enrich the subdomain alternatives (10) and (11), as illustrated in (14) and (15). This is the first step in the process of recursive exhaustification. It is schematically represented in (16).

(14) ONLY [KFP may drive the green car]
  ∼ > KFP may drive the green car, but not the orange car. ♢p ∧¬♢q
(15) ONLY [KFP may drive the orange car]
  ∼ > KFP may drive the orange car, but not the green car. ♢q ∧¬♢p
(16) 1st Exhaustification:
  a. ONLY[♢p] = ♢p ∧¬♢q
    [♢p is a subdomain alternative, with inference ¬ ♢q]
  b. ONLY[♢q] = ♢q ∧¬♢p
    [♢q is a subdomain alternative, with inference ¬ ♢p]

At the second step in the algorithm, the enriched alternatives are stacked up against the original disjunctive sentence *KFP may push the green car or the orange car*, which is cast symbolically as ♢(p ∨ q). We refer to the enriched alternatives as the “scalar alternatives” to the original assertion. The second step in the algorithm determines whether or not each of the scalar alternatives is stronger than the original statement made by the speaker. In the example under consideration, the scalar alternatives to ♢(p ∨ q) are ♢p ∧¬♢q and ♢q ∧¬♢p. If a scalar alternative is stronger than the speaker’s statement, then we make the usual inference associated with scalar implicatures; that is, we infer that the speaker was not in a position to assert the scalar alternative, so we infer the negation of the scalar alternative. It can easily be verified that both the scalar alternatives under consideration are stronger than the original disjunctive statement. The scalar alternative ♢p ∧¬♢q is stronger than the original assertion ♢(p ∨ q), and so is ♢q ∧¬♢p. Consequently, we infer their negations: ¬ [♢p ∧¬♢q] and ¬ [♢q ∧¬♢p]. This is the second step in the recursive exhaustification algorithm process. This step is represented in words in (17), and the remaining steps of the derivation are indicated symbolically in (18).

(17) ONLY [KFP may drive the green car or the orange car].
  a. ∼ > It is false that KFP may drive the green car but may not drive the orange car,
    and
  b. ∼ > It is false that KFP may drive the orange car but may not drive the green car.
(18) 2nd Exhaustification

      
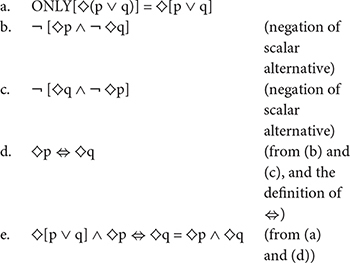


From (17-a), it follows that if KFP may drive the green car, then he may also drive the orange car (18-b), and from (17-b), it follows that if KFP may drive the orange car, then he may also drive the green car (18-c). Therefore, if he is permitted to drive either one of the cars, he is also permitted to drive the other one. Together with the original statement – *KFP may drive the green car or the orange car* – the fact that KFP may drive either car if and only if he may drive the other. (18d) entails that KFP may drive the green car and he may drive the orange car (18e). This completes our overview of the recursive exhaustification algorithm.

### *Dou* in Mandarin & Predictions for Child Language

The Q-adverb *dou* (roughly English “all”) in Mandarin Chinese is known for its multi-functions (for reviews, see Zhou and Crain, 2011; Xiang, 2020). One of the well-known functions of *dou* is an FCI licenser, which converts ∃–items, for example, pre-verbal *renhe* “any”-expressions and the non-interrogative use of *wh*-words into free choice inferences (Xiang, 2016, 2020). In view of the semantic parallelism between different ∃–items, it is pertinent to understand how the Q-adverb *dou* converts *wh*-phrases/*renhe* “any” into conjunctive interpretations, before we make predictions about the interpretation of disjunction associated with the Q-adverb *dou.* We use sentences with *wh*-words and ones with the FCI *renhe* in Mandarin as examples. When the *wh*-phrase *shenme che* precedes *dou*, as in (20), the *wh*-expression generates a conjunctive/free choice inference, just as the FCI *renhe* “any” does in (19). Both (19) and (20) have the same meaning - KFP is free to choose which of the cars to drive. If the Q-adverb *dou* is removed, however, the FCI *renhe* is no longer tolerated, resulting in the ungrammatical sentence (21). Moreover, in the absence of the Q-adverb *dou*, *wh*-phrases no longer generate free choice inferences, so example (22) can only be interpreted as a *wh*-question.

(19) Renhe che KFP dou keyi kai
   any car KFP DOU may drive
  Literal meaning: “Any car KFP DOU may drive.”
  Intended: “KFP may drive any car.”
(20) Shenme che KFP dou keyi kai
   any car KFP DOU may drive
  Literal meaning: “What car KFP DOU may drive.”
  Intended: “KFP may drive any car.”
(21) ^∗^Renhe che KFP keyi kai
   any car KFP may drive
  “KFP may drive any car.”
(22) ^∗^Shenme che KFP keyi kai
   what car KFP may drive
  “What car may KFP drive?”

The fact that *wh*-words and the FCI *renhe* are assigned a conjunctive interpretation is attributed to recursive exhaustification. A brief outline of how the recursive exhaustification algorithm derives a universal reading for the *wh*-word in sentences with *dou* is sketched in (23). We will use sentence (20) as our example.

      
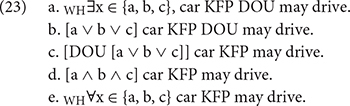


In the process, the Q-adverb *dou* plays a role, which is in analogy to the exhaustivity (ONLY) operator in recursive exhaustification algorithm (Xiang, 2016). The characteristic of the Q-adverb *dou* makes Mandarin Chinese a natural and sound laboratory to test the unified analysis of ∃–items proposed by Fox (2007) and Chierchia (2013).

The derivation of free choice inferences of *wh*-words in association with *dou* is as follows. First, *wh*-words, such as *shenme che*, are analyzed as existential indefinites. In (23), the existential expression ranges over the available cars in the domain of discourse, so we can render the meaning of the *wh*-word *shenme che* as _+ *WH*_∃x ∈ {a, b, c}. The meaning of the *wh*-word is then converted into a disjunction, [a ∨ b ∨ c], which is logically equivalent. Then, the recursive exhaustification algorithm applies, as described in the previous section. However, in this algorithm, the overt quantifier *dou* replaces the covert exhaustivity operator (only) and applies to the disjunction phrase [a ∨ b ∨ c]. The output of the algorithm is a conjunction [a ∧ b ∧ c], which is the source of the universal force attributed to the sentence, ∀x ∈ {a, b, c}.

We have seen that, in a finite domain, disjunctive phrases are the logical equivalents of the free choice item *renhe*, which logically equals to the ∃–items *wh*-words. We will see (in the literature review) that the free choice item *wh*-words receive a conjunctive/universal interpretation in sentences with *dou*. Therefore, it is a straightforward prediction that disjunction phrases in Mandarin are expected to generate a conjunctive inference when they are bound by the adverbial quantifier *dou*. This prediction was investigated in the present study. As far as we know, this prediction has not been previously verified, in either adult or child Mandarin.

To test the prediction, the disjunctive phrase must appear to the left of the Q-adverb *dou*, and there cannot be any intervening plural noun phrases^2^. These are prerequisites to the study, because *dou* is typically associated with a plural NP to its left. These conditions are satisfied in sentences like (24), where the nearest NP to the left of *dou* is the singular name, *gongfuxiongmao* “KFP.”

(24) Jiaozi huozhe shousi, gongfuxiongmao dou hui zuo.
dumplings or sushi  KFP  DOU can make
Literal meaning: “Dumplings or sushi, KFP DOU can make.”
Intended: “KFP can make dumplings and sushi.”

If the recursive exhaustification algorithm applies to (24), as expected, the following conjunctive interpretation should be a paraphrase of the meaning of (24): *KFP can make dumplings and sushi*. Mandarin-speaking children’s interpretation of sentences such as (24) will be investigated in our experiment, which we will turn to momentarily. However, before we report on the details of the experiment, it will be useful to review the findings of previous research on the acquisition of ∃–items by preschool-aged children.

## Literature Review

This section reviews previous experimental studies of the acquisition of existential expressions, focusing mainly on Mandarin Chinese. We will briefly sketch the findings of the studies that investigated children’s interpretation of three kinds of sentences: Mandarin sentences with the disjunction operator *huozhe* and the deontic modal verb *keyi* “may”; English sentences with a deontic modal verb and the FCI *any*, and its Mandarin counterpart *renhe*; and we will discuss Mandarin sentences with *wh*-words and the Q-adverb *dou*, such that we will add Mandarin *wh*-words to our stockpile of ∃–items. These studies demonstrate that 4-year-old Mandarin-speaking children draw free choice/conjunctive inferences from disjunctive statements that contain the deontic modal operator *keyi* “may” (Zhou et al., 2013), and derive free choice inferences from the non-interrogative uses of *wh*-words associated with the Q-adverb *dou* “all” (Zhou and Crain, 2011; Zhou, 2015). Moreover, 4-year-old native English and Mandarin children have acquired the dual interpretations of “any” and its Mandarin counterpart *renhe*, respectively (Tieu, 2010; Huang and Crain, 2014).

### Free Choice Inferences of Disjunction in Child Language

Zhou et al. (2013) demonstrated that 4-year-old Mandarin-speaking children (4;1-4;9, *M* = 4;3) draw free choice inferences for disjunction phrases that occur in the scope of the deontic modal *keyi* “may.” In the study, children were asked to judge sentences like (25) in a context in which KFP was only given permission to drive the green car, but not the orange car.


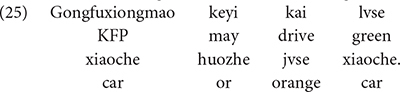

 Literal meaning: “KFP may drive the green car or the  orange car.”
 Intended: “KFP may drive the green car and the orange  car.”

If children compute a free choice/conjunctive inference for the disjunctive phrase in sentence (25), then they should judge it to be a false description of the story, because the sentence means that KFP was granted permission to drive both the green car and the orange car. Children’s judgments were exactly as predicted. Based on this finding, the authors concluded that children generate free choice inferences for disjunctive phrases that appear in the scope of the deontic modal *keyi* “may.”

### Free Choice Inferences of Any in Child Language

Tieu (2010) investigated the interpretations of *any* assigned by English-speaking preschool children (0;11,04-5;02,12), both in linguistic contexts that license NPIs and in linguistic contexts that license FCIs. To assess this, Tieu surveyed the transcripts of the spontaneous speech of 40 monolingual English-speaking children using the CHILDES database (MacWhinney, 2000). Twenty-six of the 40 children produced 15 or more instances of *any* in linguistic contexts that license NPIs with few errors. These children’s productions of *any* emerged at the same time in declarative and interrogative contexts, whereas *any* emerged significantly later in linguistic contexts that license FCIs. That is, preschool English-speaking children have the knowledge of both NPI *any* and FCI *any*.

Mandarin *renhe* behaves much like its English counterpart *any*; it can appear both in linguistic contexts that license NPIs, and in contexts that license FCIs. Huang and Crain (2014) investigated the interpretation assigned to *renhe* by 4-6 years old Mandarin-speaking children (4;5-6;3, *M* = 5;4). Sentences as in (26), in which *renhe* appeared in sentences with the modal expression *neng* “can”, and sentences as in (27) where *renhe* was omitted were tested. The finding was that the child participants assigned a conjunctive interpretation to sentences that contained *renhe*, such as (26), but not to sentences without *renhe*, as in (27). That is, children judged (26) to mean that KFP can push any one of the three cars that were made available in the experimental workspace, whereas children judged (27) to mean that KFP can only push a single car.


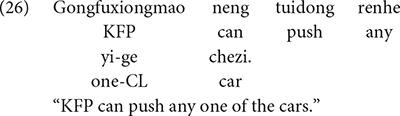



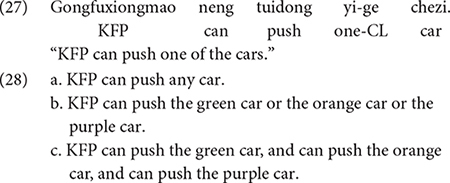


In combination with *neng* “can”, *renhe* “any” invokes a universal reading. The universal reading can be derived from an existential, such as Mandarin *renhe* “any”, by the same recursive exhausitification algorithm we described earlier. The beginning and end points of the derivation are as follows. The experimental workspace had three cars (green, orange and purple). Symbolically we can represent “*neng*… *renhe*” statements (e.g., 26) as ♢∃x ∈ {p, q, r}. This is logically equivalent to the disjunctive statement ♢(p ∨ q ∨ r), which serves as the input to the recursive exhaustification algorithm. The output of the algorithm is a conjunctive statement, ♢p ∧♢q ∧♢r, which is logically equivalent to the universal, ♢∀x ∈ {p, q, r}. In other words, the process can be represented as in (28).

### Wh-Words and the Q-Adverb *Dou* in Child Mandarin

The next series of experimental studies we review investigated children’s interpretation of Mandarin *wh*-words in combination with the Q-adverb *dou*.

The first experiment we reviewed is carried out by Zhou (2015), which assessed Mandarin-speaking children’s (3;6-4;9, *M* = 4;3) knowledge of the existential interpretation of *wh*-words in contexts that license NPIs, as in (29), and in contexts such as (30), where only the interrogative use of *wh*-words is licensed.

  
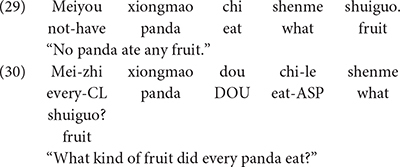


In (29), the predicate phrase of the negative (downward entailing) quantificational expression *meiyou xiongmao* “no panda” licenses the existential reading of the *wh*-word *shenme*. By contrast, the positive (upward entailing) predicate phrase of the quantificational expression *meizhi xiongmao* “every panda” in (30) does not license the existential reading of a *wh*-word. On a typical trial, three pandas were eating breakfast. All of them took one strawberry, but none of them picked a lemon. Both children and adults rejected (29) 100% of the time in this context, and they justified their responses by pointing out that every panda ate some fruit. In response to the sentence in (30), both children and adults interpreted the sentence as a question, and responded with the answer “strawberry.”

A second experiment in the same study investigated children’s understanding of the universal/conjunctive interpretation of *wh*-words in Mandarin with test sentences as in (31) and (32). For adults, the quantificational adverb *dou* is required to license the universal reading of *wh*-phrases. This is illustrated in (31) and (32). In (31), the *wh*-word *shei* “who” is bound by *dou*, thereby yielding a universal reading. By contrast, the sentence in (32) is a *wh*-question, because the *wh*-word *shei* is not bound by *dou*.

  
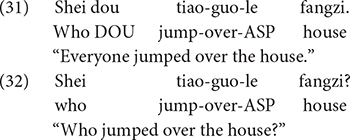


In a typical story there were three horses: a white horse, a black horse and a yellow horse. The horses engaged in a jumping competition. The white horse and the black horse easily jumped over a house, but the yellow horse was not as successful. Following the story, a puppet produced one of the test sentences; half of the children heard (31), and half heard (32). Both children and adults rejected sentenced like (31) 95% of the time, and they justified their rejections by pointing out the yellow horse didn’t jump over the house. In response to sentence (32), both children and adults consistently provided the answer to a question, usually answering “the white horse and the black horse.” The author interpreted the findings as evidence that preschool Mandarin-speaking children have the knowledge of the non-interrogative uses of *wh*-words.

Another study by Zhou and Crain (2011) investigated children’s sensitivity to the structural position of the Q-adverb *dou* and a *wh*-word using so-called *dou*-conditionals. If a *wh*-word appears in the antecedent of a *dou*-conditional, and is followed by *dou* (in the consequent clause), the *wh*-word generates a conjunctive interpretation (“whoever”). On the other hand, *wh*-words that are preceded by *dou* function as *wh*-question markers. This contrast is illustrated in (33) and (34). Although both examples contain the *wh*-word *shei* “who” and the quantificational adverb *dou*, due to these licensing conditions, the example in (33) is a statement, whereas the sentence in (34) is a question. Mandarin-speaking children (3;5-5;0, *M* = 4;3) were tested their interpretation of sentences like those in (33) and (34). In the story corresponding to (33) and (34), three villagers (a pig, a rabbit, and a dog) were being harassed by a menacing crocodile. The village head, Mr. Owl, called upon his superhero friends, Spiderman and Batman, to chase away the crocodile. After the superheroes had chased away the crocodile for the third and last time, a puppet presented (33) to one group of children, and (34) to a different group of children.

  
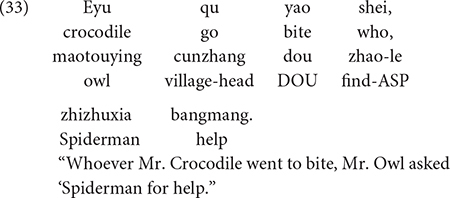


  
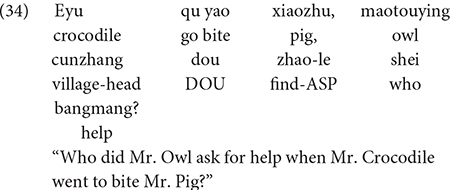


The finding was that 37 of the 42 children consistently interpreted sentences like (33) as statements, and all 42 children interpreted sentences like (34) as questions, so they responded with the appropriate answer (e.g., “Batman”). This finding provides compelling evidence that young Mandarin-speaking children know the licensing conditions on *wh*-words appearing with the quantificational adverb *dou*.

In this section, we first reviewed previous research on children’s acquisition of the existential expressions *any* in English and *renhe* “any” in Mandarin Chinese. Then we reviewed the findings of studies in which children were asked to interpret *wh*-words in sentences with the Q-adverb *dou*, versus ones without *dou* or ones in which *dou* preceded the *wh*-word. The findings demonstrated that both young English-speaking and Mandarin-speaking children have acquired the dual interpretations of a variety of existential expressions at a very early age. In the present study, we extend this line of research to the Mandarin disjunction word *huozhe*. We were interested to see whether young Mandarin-speaking children interpret the disjunctive phrases as having a conjunctive reading when they are bound by the adverbial quantifier *dou*.

## Experiment 1

This experiment was devised to assess whether or not the presence of *dou* in the sentence converts disjunctive phrases into conjunctive meanings in child Mandarin. A typical minimal pair of test sentences is illustrated in (35) and (36).

  
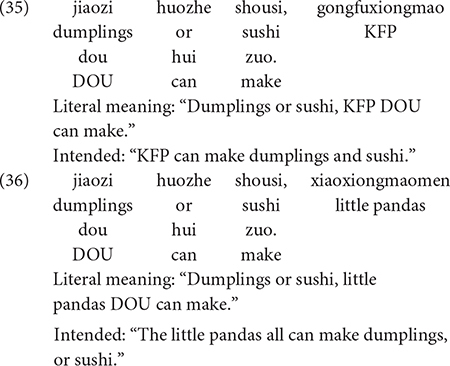


The unified account predicts that, when a disjunctive phrase occurs in the scope of the adverbial quantifier *dou*, the disjunctive phrase is converted into a conjunctive interpretation, via recursive exhausitification. This applies to sentences such as (35), where the Q-adverb *dou* takes scope over the disjunctive phrase *jiaozi huozhe shousi* “dumplings or sushi.” In this sentence, *dou* cannot take scope over the (singular) proper noun KFP, so the disjunctive phrase is the only option. Therefore, the sentence in (35) is expected to mean that KFP can make dumplings AND sushi. In (36), by contrast, there is a plural noun to *dou*’s left, *xiaoxiongmaomen* “little pandas.” Therefore, it is anticipated that the sentence means all of the pandas can make dumplings OR sushi. For brevity, we will refer to sentences like (35) as Type 1, and to sentences like (36) as Type 2.

Our experimental hypothesis is: in Type 1 sentences like (35), in which the disjunctive phrase occurs in the scope of the adverbial quantifier *dou*, children will give the disjunctive phrase a conjunctive interpretation; in Type 2 sentences like (36), in which *dou* takes scope over the plural NP, children will interpret the disjunctive phrase with disjunctive truth conditions.

### Participants

We tested 30 Mandarin-speaking children (4.18-4.90, *M* = 4.65) on their interpretation of Type 1 and Type 2 sentences. In addition, 30 Mandarin-speaking adults (18-22, *M* = 19.32) were tested as controls. All of the participants have not been reported as having developmental or psychiatric disorders, language or hearing impairments.

### Procedures

All of the adult participants and caregivers of child participants were informed of the contents and purposes of the study prior to the experiment. Adult and child participants (or their caregivers) provided written consent and oral consent before the practice trials. The procedures were in accordance with the ethical guidelines with the Declaration of Helsinki (World Medical Association General Assembly, 1964) and its later amendments or comparable ethical standards.

### Methods

The methodology we adopted was a Compute-based version of the Truth Value Judgment Task (TVJT). Originally, the Truth Value Judgment Task (TVJT) is designed to investigate the range of interpretations children assign to sentences (Crain and Thornton, 1998). The task involves two experimenters. One experimenter acts out short stories in front of child participants, using toys and props. The second experimenter plays the role of a puppet, who watches the stories alongside the child. At the end of each story, the puppet explains what he thinks happened in the story, using one of the test sentences. The child’s task is to judge whether or not the puppet said the right thing about the story. Child participants were assured that the puppet sometimes makes mistakes when he tries to describe what happened in the story. If the child judges the puppet’s statement to be incorrect, then the experimenter asks the child to explain to the puppet “what really happened” in the story. This allows the experimenters to verify that the child is rejecting the test sentences correctly. In this study, we prerecorded stories and test trials, and presented them to participants on a laptop. This helps to keep the test materials consistent between the child participants.

The child participants were introduced to the task and tested individually in a quiet room in their school. Before the test part was introduced, two practice trials were administered to familiarize the children with the task. On one practice trial, the puppet uttered a sentence that was true in the context and; on the other practice trial, the puppet said something false. This reinforced the idea that the puppet didn’t always pay attention and would sometimes say something incorrect about what had happened in the stories. Only those children who gave correct judgments to the two practice trials were included in the experiment. Adult controls were tested with a written form of the test materials.

### Materials

Eight stories were created. After each story, there were two test sentences and two filler sentences. One typical trial is used to illustrate the test scenarios of the present study.

KFP and his five little panda friends attended a cooking school. After one week of classes, they had completed the course. They came to KFP’s place to show their cooking skills.

Kung Fu master was curious to know what they could cook with only one week of lessons. He asked: “KFP, what can you make now?”

KFP: “Why don’t you guess? First, let me give you a hint. Dumplings and sushi. I can cook one of them.”

Master: “It is definitely dumplings, because your favorite food is dumplings.”

KFP nodded his agreement.

Kung Fu master went on and asked the little pandas: “Did you guys learn how to make dumplings and sushi?”

They replied: “No. It’s a shame. We did not have enough time to learn how to make both of them. All of us have learnt how to make one kind of them. We all learnt the same one. Do you know which one we can make?”

**FIGURE 1:**
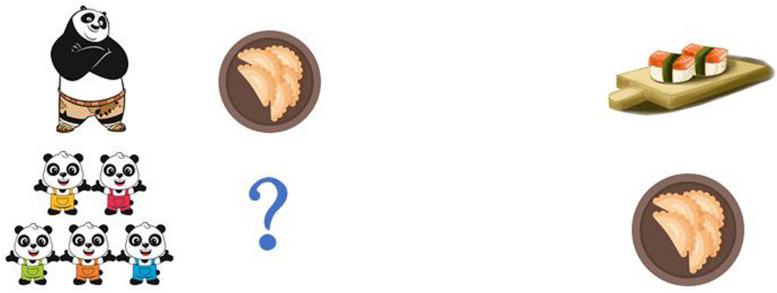
Sample test scene for sentences in (37)–(40).

The sentences in (37)–(40) were produced after the story. Example (37) means that KFP can make dumplings AND sushi. But in the corresponding context KFP can only make dumplings, participants were, therefore, expected to reject (37) by making reference to the fact that KFP cannot make sushi. By contrast, participants should accept (39), since it is a correct description of what happened in the story. To remind the participants of the events that had taken place in the story, the scene presented in Figure 1 was visible to the child participants.

  
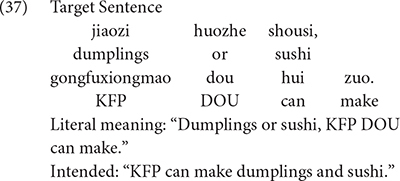


  
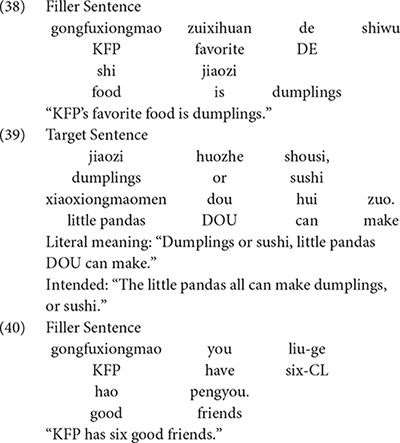


### Results

The software package of SPSS 26 was utilized to analyze the data. Percentages of correct judgments from each participant were calculated for the test trials and filler trials. All of the participants accepted the four true filler sentences, and rejected the four false filler sentences. Therefore, we did not carry out further analysis with the data of filler trials. Turning to the test sentences, children rejected Type 1 test sentences 87.5% of the time and the control group of adults rejected them at a rate of 91.7%. On the trial showcased above, for example, the children justified their rejections of (37) by explicitly referring to the fact that *gongfuxiongmao buhui zuo shousi* (“KFP cannot make sushi”) or by pointing at the sushi, meaning that KFP could not make sushi. Data analysis revealed no significant differences between children and adults in the proportion of correct responses, *p* = 0.37. In response to Type 2 test sentences, such as (39), child participants and adults accepted them 90.8% of the time and 90.4% of the time, respectively. No significant difference has been found in the proportion of the correct responses by children and adults, *p* = 0.93.

The experimental findings suggest that like adults, 4-year-old Mandarin-speaking children have the knowledge that *dou* quantifies over disjunctive phrases to its left, thereby giving disjunction phrases a conjunctive interpretation in such sentences.

## Experiment 2

This experiment, which serves as a follow-up test, was included to assess the reliability of the results of Experiment 1. Specifically, we were interested to find out whether adults’ interpretation of the test sentences would vary without the corresponding contexts in Experiment 1.

### Participants

Four groups (Group 1, Group 2, Group 3, Group 4) of Mandarin-speaking adults were tested. All of them were college students, whose age ranged from 18 to 22 and had not been reported as having developmental disorders, language or learning difficulties. Each group consisted of 63 participants. We compared the mean age of each group and found no significant difference between groups, *p* = 0.12.

### Procedures

All of the participants were informed of the purposes of the study and provided written consent to take part in the experiment. The procedures were in accordance with the ethical guidelines with the Declaration of Helsinki (World Medical Association General Assembly, 1964) and its later amendments or comparable ethical standards.

### Methods

Participants were presented audio-recorded sentences. After that, they were asked to judge the truth value of following utterances according to the firstly presented sentences. If they believe the utterances were not correct, they were required to provide their justification.

### Materials

In this experiment, each trial contained three utterances. After listening to the first utterances, participants were required to indicate whether the following utterances they heard were correct or not by putting down their answers on an answer sheet. To avoid potential carryover effect, the second and third utterances following the target sentences were presented to different groups. For example, for the utterances in (41), Group 1 heard (41a) and (41b) while Group 2 was presented with (41a) and (41c). The filler trials in (42) and (44) were presented to all of the participants.

  
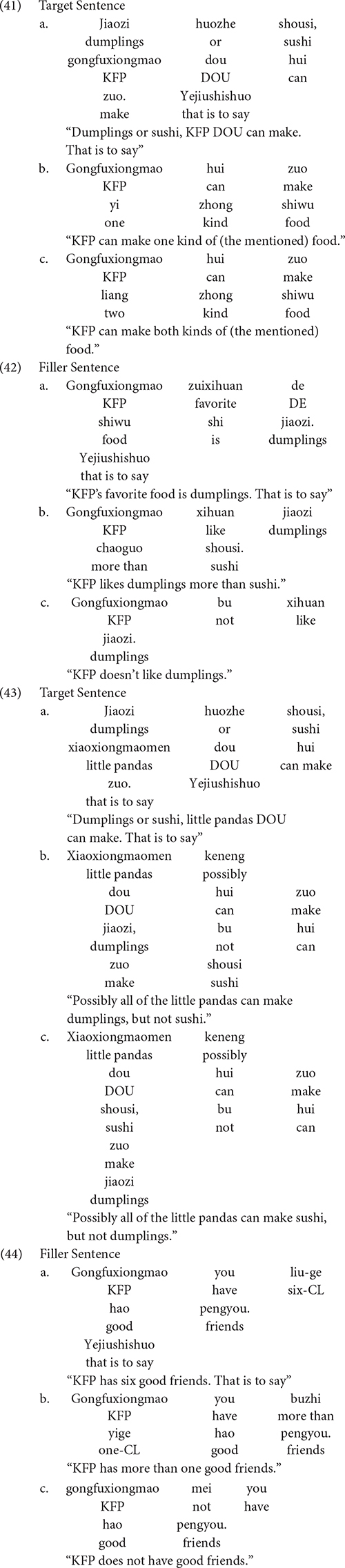


Negative answers (plus correct justifications) to (41b) and positive answers to (41c) were taken as evidence that participants assign conjunctive inferences to the disjunctive phrases in (41a). Positive answers and negative answers to (43b) and (43c) were regarded as evidence that participants assign disjunctive and conjunctive interpretations to the disjunctive phrases in (43a), respectively.

### Results

Table 1 presents the mean and standard deviation of conjunctive readings and disjunctive readings of each group. We analyzed the data with the software package of SPSS 26. Each group’s correct answer to the filler trials was above 98%, which suggested that participants were not challenged by the task. We did not analyze the results of the filler trials in the following.

**TABLE 1:** Percentage of judgments of participants in Experiment 2.

Test sentences	Group	Conjunctive reading	Disjunctive reading
Type 1 test sentence	Group 1	0.92 (0.24)	0.08 (0.24)
	Group 2	0.94 (0.22)	0.06 (0.22)
Type 2 test sentence	Group 3	0.11 (0.28)	0.89 (0.28)
	Group 4	0.05 (0.20)	0.95 (0.20)

The conjunctive readings of Group 1 and Group 2 to (41a) and the disjunctive reading assigned to (43a) by Group 3 and Group 4 were analyzed. Data analysis revealed no significant difference between Group 1 and Group 2, *p* = 0.65; and no significant difference between Group 3 and Group 4, *p* = 0.124. We interpreted our results as evidence in favor of the analysis that when the Q-adverb *dou* takes scope over disjunctive phrases, they evoke free choice inferences.

## Discussion

Previous studies revealed that, in Mandarin, *wh*-indefinites and the NPI *renhe* (English *any*) are licensed in the same downward entailing linguistic environments (e.g., in the scope of *meiyouren* “nobody”). In these environments, both *wh*-indefinites and *renhe* are typically analyzed as existential/disjunctive expressions. *Wh*-indefinites and *renhe* can also be bound by the Q-adverb *dou*, which typically takes scope over plural noun phrases to its left. When bound by *dou*, w*h*-indefinites and *renhe* generate universal/conjunctive/free choice truth conditions. This chameleon-like behavior is reminiscent of English *any*, which is as an existential item/NPI in some linguistic environments, but a universal/free choice item in others. According to one recent approach, NPI *any* and FCI *any* are uniformly existential/disjunctive expressions. In its guise as an FCI, *any* is converted from a disjunction to a conjunction by recursive exhaustification. The same, two-stage process is invoked to explain how the universal force of *wh*-indefinites bound by *dou* is derived. Specifically, an exhaustivity function ONLY first applies to a set of domain alternatives, yielding suitably enriched alternatives, and then it applies again to the output of its first application. At the second step, the exhaustivity function ONLY negates any of the enriched domain alternatives that are stronger than the original assertion. Adopting this approach leads to an interesting prediction, namely that the Q-adverb *dou* should be able to bind disjunctive NPs to its left, converting them into conjunctive interpretations.

This approach was further confirmed in the present study using sentences like (45), where the disjunctive phrase *jiaozi huozhe shousi* “dumplings or sushi” is bound by the Q-adverb *dou* (in the absence of any plural NP antecedent to the left of *dou*). The result was taken as evidence that both children and adults generated a conjunctive interpretation of (45), which can be paraphrased as KFP can make dumplings and sushi. In (46), a plural NP *xiaoxiongmaomen* “little pandas” replaced the singular *gongfuxiongmao* “KFP” in (45), such that *dou* had a plural NP to bind in (46), instead of the disjunction phrase. Consequently, the disjunctive phrase was assigned “disjunctive” truth conditions in (46), again by both children and adults. So, sentence (46) was interpreted to mean that the little pandas all can make dumplings, or sushi.

  
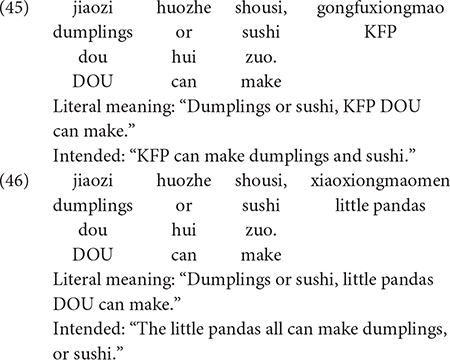


Previous work demonstrated that Mandarin-speaking children of the same age have adult-like command of sentences with *wh*-indefinites as well as the Q-adverb *dou* (Zhou and Crain, 2011; Zhou et al., 2013). Taken together, these findings invited us to conclude that, by the age of 4, Mandarin-speaking children have the knowledge of the semantics of *dou*, including recursive exhaustification. In drawing conclusions from the findings of the present study, however, it is important to be certain that the Mandarin disjunction word *huozhe* was responsible for the conjunctive interpretation assigned by children and adults.

To appreciate the potential problem, consider the sentences in (47) and (48). We will refer to (47) as a “parallel structure.” Despite the absence of the disjunction word *huozhe*, (47) generates a conjunctive interpretation, just as (48) does. The pertinent observation is that both (47) and (48) contain the Q-adverb *dou*. This raises the possibility that we would have obtained the same findings in our study if we had used sentences without *huozhe*, such as (47) instead of ones with *huozhe*, such as (48).

  
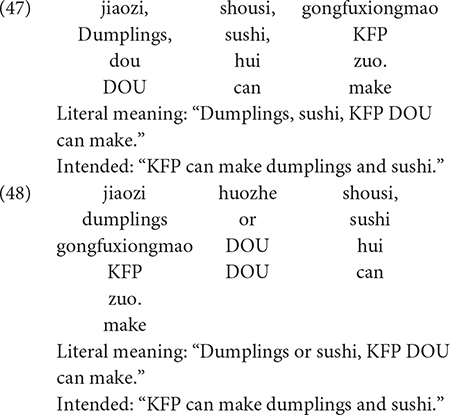


It is important to deflect the force of this potential alternative explanation of the findings. We accomplish this by showing that, although the Q-adverb *dou* is critical for deriving a conjunctive interpretation of sentences with the disjunction word *huozhe*, it is not critical in deriving the meaning of parallel structure sentences like (47).

To see that *dou* is a critical ingredient in deriving the conjunctive interpretation of sentences with the disjunction word *huozhe*, it suffices to compare sentence (48) with sentence (49), which contains *huozhe* but not the Q-adverb *dou* (as indicated by the strikethrough). Without *dou*, the disjunction phrase in sentence (49) takes on “disjunctive” truth conditions, so (49) is true if KFP can only make dumplings, or sushi. Sentence (48) would be false in this circumstance.

  
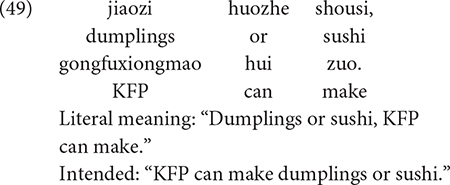


It remains to show that the Q-adverb *dou* is not critical in deriving the conjunctive reading of sentences with a parallel structure. This is accomplished using examples (50) and (51).^3^

  
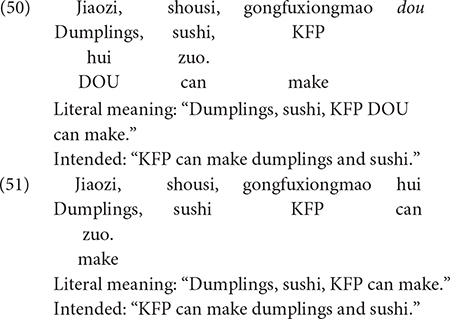


We conclude with a brief comment on how Mandarin-speaking children acquire the knowledge that they displayed in the experiment, i.e., that disjunction phrases yield a conjunctive interpretation in the scope of the universal quantifier *dou*. First, we want to deal with the possibility that children learn this from the adult input. If they do, then it should be possible to find evidence that adults produce sentences with both the disjunctive word *huozhe* and the quantifier *dou*, in sufficient abundance for children to learn the conjunctive interpretation that is assigned by adults in these sentences. To see if this acquisition scenario is on the right track, we conducted a corpus analysis of the 243,593 utterances contained in the six Mandarin Chinese corpora of CHILDES database (MacWhinney, 2000). We found no sentence with both *huozhe* and *dou*, although there were 330 sentences containing *dou* alone, and 11 sentences with *huozhe* alone. A search of another Chinese corpus TCCM (Taiwan Corpus of Child Mandarin, which includes spontaneous adult-child language samples from nine children, age ranging from 1;6 to 4;3. cf. Cheung et al., 2011) resulted in 2 instances of *huozhe* and 1143 instances of *dou*. However, there was no sentence with the combination of *huozhe* and *dou*. It should be noted that these are relatively small corpora, and that this could be a sampling error, in which case, examples of *huozhe* and *dou* could potentially co-occur in a larger corpus. Nevertheless, this low frequency of relevant input in two separate corpora makes it highly unlikely that children learn the conjunctive interpretation of disjunction based on the adult input. Therefore, we propose an alternative learnability scenario. This scenario is based on the supposition that existential items, such as disjunction words, *wh*-words, NPIs and FCIs, are innately specified in children’s grammar, as part of Universal Grammar. Following Fox (2007), Chierchia (2013), we propose that children initially analyze both *wh*-words and disjunction as existential items, and know that when *wh*-words and disjunction occur in the scope of an exhaustification operator (e.g., the quantifier *dou*), they yield a conjunctive interpretation using the kind of recursive exhaustification algorithm described in the present study.

## Conclusion

A recent proposal by Chierchia (2013) offers a unified analysis of the interpretation assigned to disjunction words, NPIs, FCIs, and the non-interrogative use of *wh*-phrases in Mandarin Chinese. Evidence in support of the analysis includes the finding that, by age four, Mandarin-speaking children interpret the existential indefinite *renhe* “any” and *wh*-words (e.g., *shenme* “what”) as NPIs when they are bound by downward entailing operators, but as FCIs when they are bound by the deontic modal *keyi* “may” or in combination with the Q-adverb *dou* “all.”

The study extends this line of research by investigating 4-year-old Mandarin-speaking children’s interpretation of the disjunction word *huozhe* “or” in sentences with *dou*, with and without an intervening plural NP. In sentences with an intervening plural NP, children assigned “disjunctive” truth conditions to disjunction phrases; but when there was a singular intervening NP, children assigned “conjunctive” truth conditions to disjunction phrases. By the age of four, then, children exhibit adult-like knowledge that disjunction phrases generate a conjunctive interpretation in sentences with the Q-adverb *dou*. This finding is taken as evidence supporting the unified analysis proposed by Chierchia (2013).

## Data Availability Statement

The original contributions presented in the study are included in the article/Supplementary Material, further inquiries can be directed to the corresponding author/s.

## Ethics Statement

The studies involving human participants were reviewed and approved by the Faculty of Human Science-Human Research Ethics Sub-Committee, Macquarie University, Australia. The ethics reference number is 5201200772. Written informed consent to participate in this study was provided by the participants’ legal guardian/next of kin.

## Author Contributions

SA and PZ conceived the study. SA designed and carried out the experiments, analyzed the data, and drafted the manuscript. SC and PZ contributed to the writing and revision of the sections “Introduction, Literature Review, Discussion, and Conclusion” part of the manuscript. All authors contributed to manuscript revision, read and approved the submitted version.

## Conflict of Interest

The authors declare that the research was conducted in the absence of any commercial or financial relationships that could be construed as a potential conflict of interest.

## Footnotes

1 The relationship between disjunction and existential quantification was made explicit in Kalish and Montague (1964). In logic texts, the symbol for disjunction is “∨.” Kalish and Montague (1964) adopted an enlarged disjunction symbol “V” to represent the existential quantifier (the symbol “∃” in other logic texts). Similarly, to capture the relationship between conjunction and universal quantification, Kalish and Montague represented the universal quantifier using an enlarged version of the conjunction symbol “∧”, namely “Λ” (rather than “∀”).

2 As noted by Xiang (2016, 2020), to license the universal free choice use of disjunction, *dou* must be followed by a possibility modal, for example, one of the Mandarin counterparts of “can.” Since the major concern of this study is with the linguistic elements preceding *dou*, we will not go into details about Xiang’s theories. Interested readers are referred to the references for more details.

3 Another example is indicted in (i). This is another parallel structure where the Q-adverb *dou* is not critical in deriving a conjunctive interpretation.  
